# Comparison of proprioception recovery following anterior cruciate ligament reconstruction using an artificial graft versus an autograft

**DOI:** 10.1186/s12891-022-06019-9

**Published:** 2022-12-03

**Authors:** Changli Xu, Tianze Liu, Miao Wang, Chang Liu, Bo Li, Qiujian Lian, Tongjiang Chen, Fengmei Chen, Suchi Qiao, Zhiwei Wang

**Affiliations:** 1 Department of Orthopedics, Changhai Hospital, Naval Medical University, Shanghai, 200433 People’s Republic of China; 2 Department of Orthopedics, The Third Affiliated to the Naval Military Medical University, Shanghai, 201805 People’s Republic of China; 3 The fifth Outpatients Department, The 980th Hospital of Joint Logistic Support Force, Shijiazhuang, 050083 People’s Republic of China; 4 Department of Orthopedics, The 900th Hospital of Joint Logistic Support Force, Fuzhou, Fujian Province 350025 People’s Republic of China

**Keywords:** ACL reconstruction, Proprioception, Artificial ligament, LARS, Sports medicine

## Abstract

**Background:**

To compare proprioception recovery after anterior cruciate ligament reconstruction (ACLR) with a hamstring tendon autograft versus the artificial Ligament Advanced Reinforcement System (LARS).

**Material and methods:**

Forty patients (9 females, 31 males) with anterior cruciate ligament (ACL) rupture were enrolled in this prospective study. Patients were randomized to two groups, 1) ACLR using a hamstring tendon autograft (*n* = 20) or 2) ACLR using artificial LARS (*n* = 20). Proprioception was assessed with knee joint position sense (JPS) passive-passive test at 45° and 75° flexions, with the contralateral healthy knee as a control baseline to calculate the JPS error. Knee JPS absolute error was used as the main outcome variable and defined as the absolute difference between the reproduction and target angles.

**Results:**

JPS error in both groups at 3 months after ACLR was significantly higher than that at 12 months. However, no significant difference in JPS error was detected between the LARS and autograft groups at either 3 or 12 months after ACLR. Analyzing JPS data by grouping patients according to whether ACLR was performed more or less than 1 year following injury regardless of graft type showed a statistically significant difference between the groups at 3 months, but not at 12 months, after ACLR. Patients receiving the graft within 1 year of injury had a lower JPS error than those receiving the graft more than 1 year after injury at 3 months. No complications were associated with either ACLR method.

**Conclusion:**

ACLR with a hamstring tendon autograft or LARS artificial graft is similarly safe and effective for recovering knee proprioception.

## Background

Anterior cruciate ligament (ACL) injury is one of the most common and devastating injuries of the lower extremity and a main cause of recurrent knee instability [1]. The ACL is necessary for static and dynamic stability of the knee joint [2, 3]. The ligament’s main role in knee joint stability is to prevent excessive anterior translation of the tibia in relation to the femur and to help trigger the “screw-home” mechanism, which occurs during femoral and tibial rotation into full knee extension [4]. Rupture of the ACL contributes to progressive functional instability and disability, which may result in secondary damage to other structures, such as meniscal tears and articular cartilage degeneration [5]. In addition, the ACL is thought to play a significant role in dynamic knee stability by affecting proprioception [4, 6].

Proprioception is the synthesis of the sensory modality of joint movement, joint position, and tactile sense encompassing a joint, whether conscious or unconscious [7]. Proprioception has been defined as the afferent information arising from the internal peripheral area of the body and contributing to postural control, joint stability, and specific conscious sensations [8]. The ACL not only provides mechanical stabilization but also contributes to proprioceptive functions, by means of the various mechanoreceptors within its structure, and at the same time, the ACL can detect changes in tension, speed, acceleration, direction of movement, and the position of the knee joint [9, 10]. Some specific ligament mechanoreceptors are found within the ACL, including the Ruffini corpuscles, Paccini corpuscles, and Golgi tendon organs, as well as a smaller number of free nerve endings that are important for proprioception [11, 12]. The proprioceptive and neuromuscular control of patients with ACL deficiency is diminished, which leads to a persistent functional instability among the injured knees and may account for an increased risk of re-injury and coordination deficits when high performance is required [13]. The decrease of sensory information after ACL injury alters the afferent information to the central nervous system, influencing sensitivity, impairing the ability to detect motion, and inhibiting muscle motor neurons that surround the joint, changing the dynamic stability of the knee [14, 15].

Proprioception is commonly assessed either with joint position sense (JPS), which is the ability to reproduce the target joint position actively or passively, or with threshold to detection of passive motion (TDPM), which is the ability to detect the initiation of passive joint movement [16]. JPS can determine where a particular body part is in space exactly by measuring the degree of angle deviation from a starting position. Muscle spindles, skin mechanoreceptors, and articular structures are likely involved in JPS [17–19]. JPS has been reported to detect a greater difference in knee proprioception than TTDPM following an ACL injury [20], and passive test procedures appear to elucidate greater differences than active test procedures [21].

There are three primary graft options for ACL reconstruction (ACLR): autograft, allograft, and synthetic graft [22]. Despite a vast amount of research, debate focused on the clinical outcomes of applying different grafts in ACLR continues. Because the hamstring tendon is the most common autograft used for ACLR, it was selected for use in the present study. Over the last few decades, synthetic grafts have been developed to avoid the need to harvest a graft from the same individual, which in itself causes musculoskeletal damage. Conceptually, these synthetic materials provide tensile strength with a maximum load to failure force that exceeds that of the native ACL, whilst limiting the complications with autograft, such as donor site morbidity, graft mismatch and prolonged periods of recuperation. The Ligament Augment Reconstruction System (LARS) is a synthetic ligament scaffold composed of polyethylene terephthalate longitudinal fibers, which are held together by transverse knitted fibers [23]. LARS is designed specifically to overcome the high percentage of mechanical failures of previous synthetic transplants and the frequently developed synovitis. Thus, LARS was selected as the synthetic graft in the present study.

Few published studies have compared knee proprioception after ACLR with an autograft and LARS. Thus, the primary aim of the present study was to longitudinally assess knee proprioception over 12 months among individuals who had undergone ACLR using LARS and to compare this with individuals who had undergone ACLR with hamstring tendon autograft. A secondary aim was to compare knee proprioception of those who had undergone ACLR within 1 year of injury with those who had undergone ACLR more than 1 year after ACLR.

## Material and methods

### Patient selection and randomization

Patients with ACL rupture were recruited for the present study from September 2018 to February 2021. ACL rupture was diagnosed based on medical history, physical examination (positive Lachman, anterior drawer, or pivot shift tests), and magnetic resonance imaging results.

The criteria for selecting patients were unilateral ACL rupture as well as patient motivation and cooperation, with or without degree I/II meniscus injury, regardless of sex or profession. Standard exclusion criteria were applied and included patients who had any of the following: combined ligament injury; radiological visible degenerative changes; contralateral knee ligament injury; articular cartilage injuries; degree III meniscus injury; chronic disease, such as diabetes mellitus; or peripheral neuropathy or vestibular dysfunction that could compromise proprioception.

All patients were informed of the benefits and risks of ACLR with the LARS (LARS; Surgical Implants and Devices, Arc-sur-Tille, France), or hamstring tendon grafts. Eligible patients assured their compliance for participation in this study and provided written informed consent. Although 61 patients met the inclusion criteria for participation in the present study, 17 of these patients did not agree to the randomization of graft selection and were excluded, 4 of these patients were lost to follow up and were excluded. Thus, 40 patients were included in the study.

**Fig. 1:**
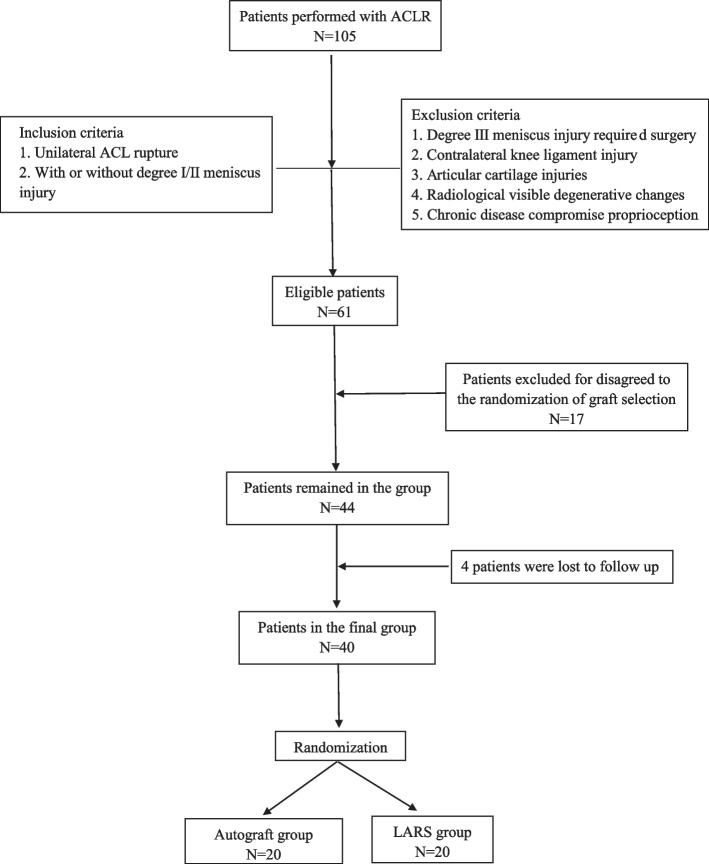
Flow chart of enrollment patients

Block randomization was accomplished with a random number generator software program (SPSS version 22; IBM Corp) to ensure equal numbers in each group. The patients were sequentially allocated into one of two surgical groups based on the results of the generated random number table. The screening process is shown in Fig. 1.

### Surgical procedures and patient rehabilitation

All procedures were assisted and performed by the same team of arthroscopic surgeons, who perform more than 100 ACLRs every year. Routine arthroscopic inspection was performed through lateral and medial infrapatellar portals using a 30° oblique arthroscope, with the knee bent at 90°. Routine arthroscopic debridement was performed on all patients, and the details are as follows.

All autograft ACLRs used the anatomic single-bundle central ACLR technique. The ACLRs were ipsilateral six-stranded semitendinosus and gracilis tendon autografts. The ACL remnants were always preserved. The tibial tunnel was drilled at a point 2 mm anterior and 2 mm medial to the center of its attachment from the medial aspect of the proximal tibia, and the femoral tunnel was centrally located between the femoral attachments of the anteromedial and posterolateral bundles of the ACL. Tunnel size and dilation were based on the composite graft size. Femoral fixation was achieved with a Rigidloop (MiTek, US), and tibial fixation was accomplished with an INTRAFIX interference screw (MiTek, US).

All ACLRs with LARS were undertaken following isometric surgical principles, which have been previously described [24]. The ACL remnants were also always preserved, and the LARS ligament was inserted through it toward the resident’s ACL ridge. The intra-articular point of the tibial tunnel was positioned at the anteromedial part of the tibial footprint of the ACL from the anteromedial tibial cortex using a tibial aimer. The tibial tunnel was drilled with a cannulated reamer matching the diameter of the graft (7.5 mm). The femoral tunnel was positioned at the 11 o’clock position on the right knee and at nearly the 1 o’clock position on the left knee. The LARS ligament was inserted into the intra-articular joint through the ACL stump, and the longitudinal free fibers were retained in the joint. This ensured that the full range of knee motion was achieved and that there was no impingement among the LARS artificial ligaments, the notch, and the posterior cruciate ligament. Afterwards, the LARS graft was fixed by two titanium interference-fit screws (LARS; Surgical Implants and Devices, Arc-sur-Tille, France).

All patients were rehabilitated using the same postoperative rehabilitation protocol for the operated knee and were assessed in a similar manner. In the protocol, the first half of the rehabilitation focused on exercises to increase the range of motion and muscle activation, while the end of the protocol focused on the training of plyometric and agility drill exercises. Quadriceps isometric closed kinetic-chain exercises and straight leg raises were initiated as early as possible after ACLR. Knee flexion began from 45° within 2 weeks after the operation and increased gradually to complete flexion and extension within 4 weeks. Thereafter, the patients stopped using crutches. The patients began to train on dynamic functional activities such as walking and double-leg squats within 8 weeks, then cycling and jogging within 12 weeks. After their first proprioception evaluation (12 weeks after ACLR), for the last part of rehabilitation, the patients began to perform neuromuscular rehabilitation exercises.

### Evaluation

The assessor performing the evaluation was blinded to the type of intervention. Data on patient age, sex, affected side, body mass index (BMI), cause of injury, and time from injury to ACLR surgery were collected. Physical examinations, including the Lachman test, pivot-shift test, and KT-1000 arthrometer measurement (MEDmetric Corporation, San Diego, CA), were performed to evaluate knee laxity. Subjective clinical assessment was obtained using the International Knee Documentation Committee (IKDC) subjective knee evaluation form, the Lysholm Knee Scale and the Tegner Activity Scale.

**Fig. 2:**
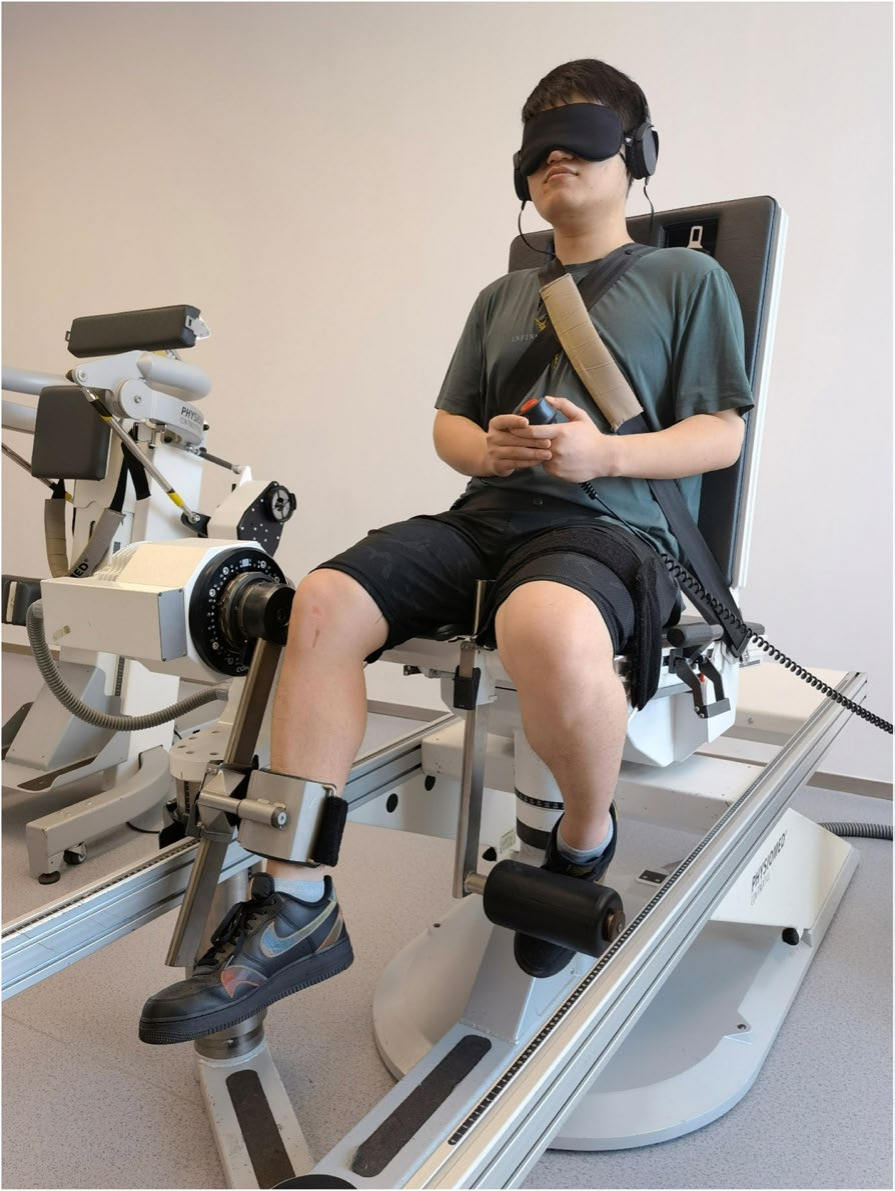
Measurement of knee joint position sense

Evaluation of proprioception was performed on both knees of all patients 3 and 12 months after ACLR using the knee JPS passive-passive test (CMV AG, Dübendorf, Switzerland) according to the modified Barrack method [17]. In preparation for the measurement, the patients were seated in a neutral angle of lumbar flexion with the popliteal fossa situated approximately five centimeters from the edge of the seat. The patient’s visual and acoustical senses were blocked with a blindfold and a headset with white noise, respectively. The JPS was measured at two angles (45° and 75°) from full extension (0°) to flexion, flexed at an angular velocity of 1°/s [17, 25]. The tested leg was flexed by a motor-driven rotational transducer interfaced with a computer to measure the reaction time of the passive motion. When the knee flexed to a predefined angle (45° or 75°), namely target angle, the leg was held in position for 5 s, and the patient was asked to remember that position, then passively returned to the starting position. The target angle procedure was repeated twice. The knee was returned to the starting position and then moved again by the motor at the same speed. When the patient felt that the leg was in the same position as before, the patient stopped the movement of the machine with a switch. The deviation between the knee angle at the time of the button press and the target angle (angular absolute error) was measured. The JPS error of both knees was measured three times for every patient, with 1 min rest at each interval and the mean value of the data produced three times was considered the final data. Given that each patient may have a different proprioception baseline, each patient’s contralateral knee was used as the baseline to calculate the JPS errors (Fig. 2).

### Statistical analysis

Power analysis was performed using G*Power 3.1(Heinrich Heine University Düsseldorf, Germany) to determine the effect sample size. The effect size was calculated according to the data in our prior study. For two-tailed analysis with the power of 0.90, with α = 0.05, the estimated size of each group was 12 at minimum. Statistical analyses were performed using SPSS (version 22; IBM Corp). The Shapiro-Wilk test was used to access the normal distribution of the data，and Levene’s test was used to evaluate whether there was equal variance in the continuous variables between the two groups. The Fisher’s exact test was used to assess categorical variables. Non-parametric tests (Mann-Whitney U test) were used to assess Lysholm Scale score, IKDC score and Tegner score, and Student’s t-test was used to assess continuous variables with normal distributions (including Paired t-test and Unpaired t-test). A two-sided *P* < 0.05 was considered statistically significant.

## Results

### Results by grouping patients according to graft type

Forty patients with ACL rupture participated in this study, which were beyond the required size. The autograft group using a six-strand ipsilateral hamstring autograft (semitendinosus and gracilis tendons) included 20 patients, 14 males and 6 females. The LARS group included 20 patients, 17 males and 3 females. Demographics and preoperative parameters are shown in Table 1. There were no significant differences between the groups for the number of patients, sex distribution, age, BMI, Lysholm Scale score, IKDC score or Tegner Activity Scale. However, the time between the injury and ACLR for the autograft group was significantly longer than that for the LARS group.

**Table 1:** Demographic and clinical characteristics of participants

Characteristic	Autograft group (*n* = 20)	LARS group (*n* = 20)	*P* value
Age (years)	37 ± 12 (20–51)	36 ± 10 (17–48)	NS^a^
Male/Female(n)	14/6	17/3	NS^c^
BMI (kg/m^2^)	24.3 ± 4.7	24.9 ± 5.7	NS^a^
Lysholm Scale score	52.5 ± 10.5	54.5 ± 8.7	NS^b^
IKDC score	47.8 ± 5.7	51.2 ± 8.6	NS^b^
Tegner score (pre-operation)	2 (1–3/1)	2 (1–2/1)	NS^b^
Tegner score (12 months post-operation)	3 (2–5/0)	3 (3–4/0)	NS^b^
Time from injury to ACLR operation (months)	18.4 ± 8.5	10.8 ± 6.1	0.002^a^

Data were expressed as mean ± SD (range) unless otherwise indicated, Tegner scores were presented as median (range / IQR); *ACLR* Anterior cruciate ligament reconstruction, *BMI* Body mass index, *IKDC* International Knee Documentation Committee, *SD* Standard deviation, *IQR* Interquartile range, *NS* Statistically non-significant

^a^Calculated by Unpaired t-test. ^b^Calculated by Mann-Whitney U test. ^c^Calculated by Fisher’s exact test for categorical variables between two groups

Significantly greater JPS errors were found for ACLR knees compared with contralateral asymptomatic knees. There was no significant difference in the JPS errors between the LARS group and the autograft group at either 3 or 12 months after the ACLR operation in the 45° or 75° flexion JPS test. However, for both the 45° and the 75° flexion JPS tests in both groups, the JPS errors were significantly greater at 3 months after ACLR than at 12 months after ACLR (Table 2).

**Table 2:** Knee JPS errors (°) in the autograft and LARS groups 3 and 12 months after ACLR

Time of evaluation (months)	45° flexion			75° flexion		
	3	12	Statistic	3	12	Statistic
Autograft group	2.81 ± 1.41	1.87 ± 0.96	0.001^a^	2.74 ± 1.13	1.77 ± 0.79	0.016^a^
LARS group	2.16 ± 0.85	1.65 ± 0.73	0.022^a^	2.42 ± 0.79	1.73 ± 0.56	0.028^a^
Statistic	NS^b^	NS^b^		NS^b^	NS^b^	

Data were expressed as mean ± SD (range) unless otherwise indicated; *ACLR* Anterior cruciate ligament reconstruction, *LARS* Ligament Advanced Reinforcement System, *SD* Standard deviation, *NS* Statistically non-significant

^a^Calculated by Paired t-test. ^b^Calculated by Unpaired t-test

### Results by grouping patients according to the time from injury to operation

During our study, we noticed an interesting finding that there was a difference of the JPS errors in patients with their time from injury to operation. Therefore, we reanalyzed the data by grouping the patients according to the time from injury to operation as more than 1 year (18 patients) and less than 1 year (22 patients). There were no statistically significant differences between these two groups in the number of patients, sex distribution, age, BMI, type of surgery, Lysholm Scale score, IKDC score or Tegner Score (Table 3).

**Table 3:** Demographic and clinical patient characteristics analyzed by time from injury to ACLR more or less than one year

Characteristic	Time from injury to ACLR operation(years)		*P* value
	> 1(*n* = 18)	< 1(*n* = 22)	
Age (years)	35 ± 10 (17–49)	37 ± 12 (20–51)	NS^a^
Male/Female(n)	13/5	18/4	NS^c^
BMI (kg/m^2^)	25.1 ± 4.3	24.8 ± 4.9	NS^a^
Type of surgery: autograft/LARS (n)	11/7	9/13	NS^c^
Lysholm Scale score	47.2 ± 8.3	54.1 ± 9.6	NS^b^
Tegner score (pre-operation)	2 (1–2/1)	2 (1–3/1)	NS^b^
Tegner score (12 months post-operation)	3 (3–4/0)	3 (2–5/0)	NS^b^
IKDC score	49.8 ± 16.3	52.6 ± 18.7	NS^b^

Data were expressed as mean ± SD (range) unless otherwise indicated, Tegner scores were presented as median (range / IQR); *ACLR* Anterior cruciate ligament reconstruction, *BMI* Body mass index, *IKDC* International Knee Documentation Committee, *SD* Standard deviation, *IQR* Interquartile range, *NS* Statistically non-significant

^a^Calculated by Unpaired t-test. ^b^Calculated by Mann-Whitney U test. ^c^Calculated by Fisher’s exact test for categorical variables between two groups

A statistically significant difference was found between the groups with more and less than 1 year from injury to ACLR at both the 45° and 75° flexion JPS tests 3 months after ACLR; however, there was no significant difference between these groups for these measures 12 months after ACLR. But the JPS errors from both the 45° and 75° flexion JPS tests for both groups 12 months after ACLR were significantly lower than those 3 months after ACLR (Table 4).

**Table 4:** Knee JPS errors (°) in patients analyzed by more or less than 1 year from injury to ACLR, assessed 3 and 12 months after ACLR

Time of JPS evaluation (months)	45° flexion			75° flexion		
	3	12	Statistic	3	12	Statistic
ACLR more than 1 year after injury	3.15 ± 1.05	1.96 ± 1.13	0.001^a^	2.72 ± 1.04	1.85 ± 0.02	0.013^a^
ACLR less than 1 year after injury	1.76 ± 0.72	1.45 ± 0.87	0.032^a^	1.80 ± 0.87	1.37 ± 0.54	0.025^a^
Statistic	0.016^b^	NS^b^		0.029^b^	NS^b^	

Data were expressed as mean ± SD (range) unless otherwise indicated; *ACLR* Anterior cruciate ligament reconstruction, *JPS* Joint position sense, *NS* Statistically non-significant

^a^Calculated by Paired t-test. ^b^Calculated by Unpaired t-test

At the one-year follow-up examinations, the results measured using the KT-1000 arthrometer showed that over 90% of the patients in both groups had side-to-side differences of 2 mm or less. In addition, the lateral pivot shift test showed no significant difference between the two ACLR groups (all were negative). However, this measure had improved compared with that obtained preoperatively. In the final test at the one-year follow-up, the mean Lysholm Scale scores between the autograft (91.3 ± 3.8) and LARS (90.6 ± 5.2) ACLR groups were not significantly different; however, compared with their preoperative Lysholm Scale scores, all patient Lysholm Scale scores had significantly improved (*P* < 0.05). There was no significant difference between the groups either in preoperative or postoperative Tegner scores; however, all patient postoperative Tegner scores had significantly improved (*P* < 0.05) compared with their preoperative ones. Moreover, all patients achieved full range of motion, and there were no complications, including no infection or early graft failure, within 12 months of ACLR.

## Discussion

In this study, we found there was no significant difference in proprioception recovery between the LARS and the autograft groups at either 3 or 12 months after ACLR. Another finding was that a significant difference in proprioception was found between the groups with more and less than 1 year from injury to operation at 3 months after ACLR; however, there was no significant difference between these groups at 12 months after ACLR. The results revealed that proprioception in all patients regardless of graft type or the time from injury to operation could be recovered to same level after rehabilitation exercises.

Proprioception is considered a key element in sensorimotor control [26]. The proprioceptive deficit of a knee with a ruptured or nonfunctional ACL is well documented [27–31]. As a specific sub modality of proprioception, knee JPS test is most often used to assess knee proprioception. JPS test procedures include active-active, passive-active and passive-passive, regarding weight-bearing or non-weight-bearing. Within studies, JPS tests were considered separate if they differed regarding modifiable components of reproduction method, direction of movement, body position, measurement equipment, test procedure and different target angles [21]. However, there is no standardized knee JPS test. A recent systematic review and meta-analysis by Strong et al. found that the discriminative validity and sensitivity of knee JPS tests targeting individuals with ACL injury was rated as sufficient and passive test procedures appeared more sensitive than active test procedures [21]. The data of the present study was based on absolute error of knee JPS passive-passive test. It should be noted that absolute error was the sole outcome measure considered in this study as it was by far the most commonly reported. However, constant and variable error may provide valuable information regarding JPS and may provide different results when compared with absolute error. Further investigations focusing on the potential effects of these factors, such as angular error, different test procedures and weight-bearing or non-weight-bearing tests, would be of clinical and scientific value.

Meta-analyses have found significantly poorer knee proprioception among ACL-injured knees [21]. Much more attention has been paid to proprioception related to rehabilitation and gait than to the surgery associated with the injury. To improve long-term function, physicians have established rehabilitation programs for patients that focus not only on range of motion and strengthening exercises but also on proprioceptive and neuromuscular control drills [20, 32–34]. Müller et al. showed that a functional neuromuscular training program was more effective than traditional muscle strength rehabilitation programs in improving function [34]. The results from all these aforementioned studies indicated that appropriate proprioception exercises would be expected to achieve significant improvements in knee proprioception and function in patients following ACLR.

The present study found no significant difference in the loss of proprioception between the LARS and the autograft groups 3 months after ACLR. The proprioception of patients in both groups 12 months after ACLR was significantly improved after rehabilitation exercises. The present study also found no significant difference in proprioception and the data of the KT-1000 arthrometer measurement, pivot shift test and patient-reported outcomes between the two graft groups 12 months after ACLR. These results indicate that ACLR with either an autograft or a LARS artificial graft is safe and effective and that proprioception in patients with a LARS artificial ligament could be recovered to same level as that in patients with an autograft after rehabilitation exercises.

Previous studies have suggested that the knee proprioception deficit was associated with the disruption of mechanoreceptors within the ligament and subsequent loss of proprioceptive feedback [35, 36]. However, Sha et al. used immunohistochemical assays to show that there was no significant quantitative variation in the residual mechanoreceptors throughout the injury duration [37]. Another study showed that the mechanoreceptors existed in the cruciate ligaments, articular capsule, the lateral structures such as the lateral collateral ligament and the anterolateral ligament, tendons and muscles of the knee [8]. Trieb et al. found that instead of normal ligament tissue 6 months after ACLR with LARS, there were only fibroblasts and some endothelial cells surrounding the ligament fibers [38]. Therefore, after ACL rupture, the knee proprioception deficit may have a closer relationship with the abnormal neurologic output from the articular capsule, muscle, and other soft tissue than with the ACL itself. The results of our study indicated that proprioception recovery in the LARS group was similar to that in the autograft group. Therefore, the knee proprioception recovery after ACL rupture may be related primarily to the compensation of articular capsule, muscle, and other soft tissue.

The analysis of the data based on the time from injury to ACLR showed that patients with more than 1 year from injury to operation had a greater JPS error 3 months after the ACLR than those with less than 1 year from injury to operation. This result indicated that early treatment after injury may improve postoperative knee function sooner. A recent meta-analysis by Kosy et al. showed a decrease in the mechanoreceptors in the remnants of the ruptured ACL with increasing time from rupture in multiple histological studies [10]. Nevertheless, 12 months after ACLR, there was no significant difference in proprioception between these two groups, indicating that knee proprioception in patients could substantially recover following rehabilitation exercises 12 months after ACLR. From a physiological point of view, with time going on, knee proprioception improvement may be due to higher order central nervous system (CNS) adaptations to the peripheral signals from muscle spindles and joint receptors at the slow or fast velocities [39].

Certain limitations of the present study should be addressed. First, only 40 participants were recruited, which limited us to use more complex statistical models, incorporating the time from injury as a covariate to explore the integration between this and the type of intervention. Second, it is not easy to track such a long period and follow the individual variability in the physiotherapy sessions while at home, though all the patients achieved a satisfactory functional performance. Third, the follow-up time was limited to 1 year, and it might be speculated that time-dependent changes in proprioception occur caused by histological healing processes. With larger cohorts and multiple follow-up evaluations, additional studies will be needed to observe the long-term effects of ACLR with different grafts on proprioception.

## Conclusion

We found no significant difference in proprioception recovery between patients receiving a LARS graft and those receiving a hamstring tendon autograft 3 or 12 months after ACLR. No complications, including infection or early graft failure, were associated with either ACLR method. These results suggest that ACLR with either an autograft or a LARS artificial graft is similarly safe and effective for knee proprioception recovery.

## Data Availability

The data contributing to this article may be made available upon request by sending an e-mail to Changli Xu or Tianze Liu.

## Abbreviations

ACL: Anterior cruciate ligament
ACLR: Anterior cruciate ligament reconstruction
LARS: Ligament Augmentation and Reconstruction System
JPS: Joint position sense
CNS: Central nervous system
TDPM: Threshold to detection of passive motion
BMI: Body mass index
IKDC: International Knee Documentation Committee
SD: Standard deviation
